# Family caregiving during the COVID-19 pandemic: factors associated with anxiety and depression of carers for community-dwelling older adults in Hong Kong

**DOI:** 10.1186/s12877-021-02741-6

**Published:** 2022-02-14

**Authors:** Marcus Y. L. Chiu, Cyrus L. K. Leung, Ben K. K. Li, Dannii Yeung, T. W. Lo

**Affiliations:** 1 Department of Social and Behavioural Sciences, City University of Hong Kong, Kowloon, Hong Kong; 2 University of Bolton, England, UK; 3 JC School of Public Health and Primary Care, The Chinese University of Hong Kong, New Territories, Hong Kong

**Keywords:** Family carers, Depression, Anxiety, Hong Kong, COVID-19 pandemic

## Abstract

**Background:**

The COVID-19 pandemic disrupts the daily routine and increases the caregiving load of the family carers of older adults. This study examined how the pandemic may impact mental health and investigated the prevalence of anxiety and depressive symptoms in family carers of older people.

**Methods:**

Two hundred and thirty-six family carers of older adults participated in this cross-sectional survey study. Outcome measures included their symptoms of anxiety and depression, pandemic-related psychosocial factors, external factors, and the practice of preventive behaviours.

**Results:**

Caseness prevalence of anxiety and depression among family carers was 25 and 56% respectively. Working carers were more depressed than non-working, while younger carers reported more anxiety and depression symptoms than older. Hand hygiene and getting drugs from the hospital positively predicted depression while healthy lifestyle negatively predicted depression. These variables, together with perceived risk and severity and the worry of getting infected, predicted anxiety.

**Conclusions:**

The prevalence of mental health symptoms was alarming. Telemedicine practice, including online pharmaceutical services and the Internet Hospital plus Drug Delivery platform, could be a solution in alleviating the burden and worry of infection of family carers. Tailored-made interventions by age and working status of the family carers are recommended.

## Introduction

Caregiving for older adults is particularly challenging in the 2019 Coronavirus Disease (COVID-19) pandemic. In Hong Kong, the government and non-government organizations have offered various community care and support services to community-dwelling older adults, including the elderly health centres and geriatric day hospitals specialized in dealing with problems of needed older adults [1–3]. The needs-based services become possible to carry out geriatric assessments, continued care, health education and promotion, counselling, and rehabilitative services that tailor for the need of community-dwelling older adults [1, 4]. To impede the transmission among older adults during clinic visits, there was restricted access to these health and community services under the social distancing measures [5, 6]. Non-essential medical and social services were suspended [3, 7], and the attendances of out-patient clinics and health services significantly dropped [6, 8, 9]. Access to medications was impeded [8]. Health systems in Hong Kong and other countries depend on family carers to provide care to older adults more than ever [5, 10]. Family carers are experiencing substantial pressure with the increased demands on caregiving and restrictions on household chores at this critical moment [11].

Carers of older adults in Hong Kong families are usually the spouse or adult child, with two-thirds of these carers of age 60 or above in a population survey conducted in 2018 [12, 13]. Older family carers would find it stressful and disruptive in affording the more demanding care of their spouses, as older adults are more susceptible to COVID-19 infection and vulnerable to adverse clinical outcomes of the infection [14, 15]. Not only the COVID-19 pandemic is most deadly for older adults, but the uncertainty and threat brought by the pandemic are also detrimental to the mental health of these older adults. Studies conducted in Spain, Italy, and the United Kingdom reported that older adults with chronic illness and their family carers suffered from higher stress, anxiety, and depressive symptoms [16–18].

To mitigate the spread of the virus, the Hong Kong government enacted social distancing and health quarantine measures [19], including quarantine procedures for entry via the airport, restrictions on dining in at restaurants (restrictions on opening hours, a capped maximum of the number of people for each table), the prohibition of group gatherings (which restricted many of social activities including family gatherings), closure or restricted capacity of certain kinds of business (including gyms, beauty parlours, pools, spas, cinemas, theme parks, etc.), and adjustment or curtailment in health services (suspension of geriatric day-hospital services, day-care centre services and outreach rehabilitation services; restrictions of visiting in the hospitals). Although the social distancing and health quarantine measures are deemed effective in containing the disease, these changes in the COVID-19 pandemic are putting increasing demands on family carers in providing daily care and support beyond their usual caregiving responsibility with deprived social support [17], which are detrimental to the mental health of the family carers.

The fourth wave of the pandemic arrived in Hong Kong in late November 2020, and the vaccine would not be available for the public until the first quarter of 2021 at the earliest [20]. For the increased reliance on family carers in taking care of the community-dwelling older adults, protecting family carers are crucial to the healthcare systems as a whole in preparing subsequent waves of outbreak and future pandemics. It is imperative to examine the impact of the quarantine and social distancing measures on the psychological and mental health of the family carers of older adults (RQ1), and thus to inform about the need of the family carers. In the current study, we set out to examine the correlates of the COVID-19 pandemic on the family carers of older adults in three folds: the psychosocial factors, the external factors, and the practice of preventive measures.

### Psychosocial factors

Perceived risk and severity are central appraisal constructs in conventional health behaviour models such as the Health Belief Model [21], Protection Motivation Theory [22], and Health Action Process Approach [23], with anxiety and fear as common emotional consequences. The psychosocial impact of the COVID-19 pandemic does not only come with the risk of infection but also with the risk of unemployment and other disruption of economic activities. The COVID-19 pandemic has had a substantial impact on the global economy, as demonstrated by the increase in the worldwide rate of unemployment. The perceived employment and financial instability or difficulties are bringing anxiety and are detrimental to mental health [24]. Family carers are by no means exempted from these economic conditions or their mental health implications. We expect these psychosocial factors related to the risks brought by the COVID-19 pandemic would contribute to anxiety and depression among family carers.

### External factors

The changing external environment also makes the caregiving burden heavier than ever. Family carers have to protect themselves and the older adults from COVID-19, making them reluctant to expose themselves to risky healthcare settings [25, 26]. They have also reported worry about the storage of daily supplies [11]. At the time of data collection, citizens were keen on the demand for masks, and thousands of Hong Kong people had to queue up outside drugstores and supermarkets for masks amid shortages [27]. Suspension or curtailment of non-emergency healthcare services and scarcity of healthcare resources delayed access to healthcare support and necessary medical treatment [28, 29]. All these may easily increase the anxiety level of the family carers. Besides the disruption of the caregiving routine of the family carers, these pragmatic challenges place dilemmas on the family carers whether to expose themselves and the older adults to the risk of infection or to delay treatment and services, of which may deteriorate the physical and mental conditions of the older adults. For older adults who were put in residential care, recent pandemic outbreak in nursing homes may make family carers both worried and helpless when they were denied access because of intensive preventive measures. Situations in private nursing homes are particularly worrying as inadequate manpower of private nursing homes in pre-pandemic time relied much on family carers to supplement a helping hand to routine care such as rolling over and massage to prevent bedsores. Frail older adults whose caregiver is now denied entry, would also mean a denial of a necessary service and emotional support to the older adults, and the creation of a helpless situation where a family caregiver can do nothing about it.

### Protective measures

The lockdown or social distancing measures, being the essential interventions to mitigate the transmission, are disruptive to the very social interactions that are essential for good mental health and hampering social support for better coping [30]. Social distancing measures also contribute to the sense of loneliness [26, 31] and bring substantial impact on mental health, including generalized anxiety and depression in the case of true quarantine [32]. The relationship of other protective measures, including wearing a mask, hand hygiene and living a healthy lifestyle, are less clear with anxiety in the literature. We would explore the effect of these social distancing measurers on the mental health of the family carers.

This study also set out to examine the age difference of coping in this difficult time among the family carers (RQ2). According to the socioemotional selectivity theory, there is a difference in goal orientation between younger and older adults and an age-related increase in the selection of positive and emotionally meaningful memory [33, 34], and that bring differences in regulation of emotions, processing positive and negative information and coping. We would expect older family carers would be better in regulating their negative emotions in their caregiving role and hence report less anxiety and depression symptoms during the pandemic.

To summarize, the aim of the current study is to look into the common mental health issues of anxiety and depression among family carers during the pandemic period and to ascertain its associated factors (RQ1); and to examine the demographic difference of mental health among family carers (RQ2).

## Methodology

### Power analysis

We used hierarchical regression analyses in examining the predictors of anxiety and depression among family carers. We calculated the required sample size using G*Power version 3.1. Considering an expected small effect size (around .10), 15 predictors to be included in the models, a power level at .95, and an error probability level at .05, the required sample size to detect the effect would be 133. The current valid sample size (*N* = 236) is sufficient to detect the potential effect.

### Participants

Between April and May 2020, 386 family carers were invited through four local non-governmental organizations (NGOs) from different districts in Hong Kong, and 236 were recruited, with a response rate at 61.1%. The mean age of the participants was 62.68 (*SD* = 14.93), with three-fourths being women (*n* = 175). Around 60% of the family carers had a full-time or part-time job (*n* = 143). The mean year in caregiving was 7.44 years (*SD* = 7.51). The current sample was representative, and its demographic characteristics were similar to a recent community survey in 2018 assessing caregivers’ needs [35] (*N* = 1115, *M*_age_ = 65.28, *SD*_age_ = 15.07; percentage of female = 78.6%),

### Procedures

After giving informed consent, participants completed the survey themselves either online or with a hardcopy questionnaire distributed by the service units. For those who have difficulty accessing online means or problems of comprehension due to level of literacy, they will be given a telephone interview, depending on their preferences. On average, it took around twenty minutes to complete the online survey or telephone interview, and an HKD$50 supermarket voucher was given as an appreciation of their participation.

### Measures

#### Psychosocial factors

##### Perceived risk and severity

Perceived risk and severity of infection were measured using a five-item scale, which was used to investigate the perceived risk and the worries about COVID-19 infection in both healthcare workers and the general population in Italy [36]. There are two items measuring perceived risk (Cronbach’s α = .88) and three items for perceived severity (Cronbach’s α = .84). To better fit our purpose, we modified the wording of the item “Did you think you were at risk when the first cases appeared in Italy in January 2020?” to “Did you think you were at risk of being infected recently?”. Participants answer this 5-item scale on a 5-point Likert scale ranging from 0 (never) to 4 (always). The higher mean score of the scale indicates the greater perceived risk and severity of the infection.

##### Perceived financial strain

Participants answered two dichotomous items developed by the research team (1 = *yes*; 0 = *no*) on their financial (“The pandemic has bought financial difficulty to my family in covering the rent and food expenses”) and employment difficulties (“The pandemic makes me or my family members losing the job”).

#### External factors

##### Protective supplies

We also investigated the family carers’ perceived sufficiency of preventive materials for the COVID-19 outbreak, including adult masks, children’s masks, alcohol hand rub, hand soap, bleach, disinfectant wet wipes, and other epidemic prevention equipment, such as an eye shield. Participants reported their amount of stockpile and rated on a 5-point Likert scale ranging from 1 (very insufficient) to 5 (very sufficient). A higher mean score indicates higher perceived sufficiency of preventive materials. The scale has good reliability in our sample (Cronbach’s α = .91).

##### Other external factors

Four dichotomous items were developed to assess the external factors that disrupt the life of the participants and their families, including home infection risk, suspension of community centre services, getting drugs from the hospital, and delayed treatment at the hospital (1 = *yes*; 0 = *no*).

#### Preventive Measures

##### Social distancing

Social distancing practice was measured using a five-item scale developed by Oosterhoff and Palmer [37]. This scale was used to examine the behaviours of participants in the United States during the COVID-19 outbreak. Participants indicated how often they spent time in the past 7 days in person with their friends, colleagues, neighbours, extended family, and any other person who are not living in the same place on a 5-point Likert scale from 1 (not at all) to 5 (very often). We reverse coded the items, and a higher mean score indicates more social distancing practice. The reliability of the scale is good (Cronbach’s α = .74) in our sample.

##### Other preventive behaviours

Preventive behaviour was assessed using the SARS-Preventive Behaviours scale [38]. This scale was used in a study that examined the preventive behaviour in people from the Severe Acute Respiratory Syndrome (SARS)-affected regions included Guangdong, Hong Kong, Singapore, and Toronto in the SARS epidemic in 2003. Since both COVID-19 and SARS are caused by coronaviruses and are airborne infectious diseases, they share the same preventive measures [39]. We used items related to wearing a mask (one item), hand hygiene (two items), and a healthy lifestyle (three items) in our study. One additional item, “Wash your hands for at least 20 seconds”, was developed for hand hygiene in response to the recommendation from the Department of Health of Hong Kong. Participants rated on a 5-point Likert scale ranging from 1 (very unlikely) to 5 (very likely). A higher mean score indicates a higher reported frequency of performing a particular COVID-19 preventive behaviour. The hand hygiene subscales (Cronbach’s α = .69) and healthy lifestyle (Cronbach’s α = .67) had acceptable reliability in our sample (Table 1).

**Table 1:** Descriptive Statistics on Demographics and Measurements (*N* = 236)

	*Mean* / %	*SD*	Possible range	Cronbach’s α
Demographic factor				
Age	62.68	14.93	> 18	–
Women	74%	–	0–1	–
Working	61%	–	0–1	–
Year in caregiving	7.44	7.51	> 0	–
Psychosocial factor				
Perceived risk	2.68	0.87	1–5	.88
Perceived severity	3.18	0.94	1–5	.84
Employment difficulties	36%	–	0–1	–
Financial difficulties	30%	–	0–1	–
External factor				
Protective supplies	3.76	0.70	1–5	.91
Home infection risk	11%	–	0–1	–
Center services suspended	25%	–	0–1	–
Getting drugs from the hospital	28%	–	0–1	–
Delayed treatment at the hospital	19%	–	0–1	–
Protective measure				
Wearing mask	4.86	0.41	1–5	–
Hand hygiene	4.03	0.74	1–5	.69
Social distancing	3.64	0.85	1–5	.74
Healthy lifestyle	3.42	0.71	1–5	.67
Mental health outcome				
Anxiety	5.36	3.22	0–21	.84
Non-case (0–7)	75%	–		
Mild (8–10)	19%	–		
Moderate (11–14)	5%	–		
Severe (15–21)	1%	–		
Depression	8.35	3.33	0–21	.77
Non-case (0–7)	44%	–		
Mild (8–10)	30%	–		
Moderate (11–14)	22%	–		
Severe (15–21)	4%	–		

#### Mental Health Outcomes

Anxiety and depression were measured using the Hospital Anxiety and Depression Scales (HADS) [40]. HADS has been used in studies investigating the psychological responses regarding the COVID-19 pandemic in Iran and Hong Kong [41, 42]. This scale has good reliability (Cronbach’s α > 0.8) in the general population, practice patients, medical patients, and psychiatric out-patients [43]. Half of the items in this 14-item scale tapped into depression (HAD-D) and another half into anxiety (HAD-A). Participants replied on a 4-point scale (from 0 to 3) for each item, and the possible total score range for both anxiety and depression is from 0 to 21, respectively. Sample items included “I get a sort of frightened feeling as if something awful is about to happen” for HAD-A and “I have lost interest in my appearance” for HAD-D. The reliability of HAD-A and HAD-D were .84 and .77 in our sample, respectively. In assessing the symptom severity and finding possible cases of emotional disorder, a threshold of 8+ is optimal in terms of sensitivity and specificity for both depression and anxiety [44], while 11+ would be the indication of moderate or severe cases [45]. This paper followed the conventional classification of four different levels of severity for both anxiety and depression: Non-case (0–7), Mild level (8–10), Moderate (11–14), and Severe (15–21).

## Results

### Psychosocial factors

The descriptive statistics of the studied variables are shown in Table 1. About 30% of the participants reported general financial difficulties, and more than one-third (36%) had a problem with employment during the pandemic time. Considering the perception towards COVID-19 pandemic, the mean score of perceived risk and perceived severity were 2.68 (*SD* = 0.87) and 3.18 (*SD* = 0.94), respectively, on a 5-point scale (0–4).

### External factors in concern

Participants, in general, found they had more or less sufficient protective supplies, with the mean score at 3.76 (*SD* = 0.70). One-quarter experienced the suspension of centre services; nearly one-fifth had delayed treatment from the hospital, and more than a quarter had the need to get medications from the hospital (28%). The percentage of participants raising concern about delayed treatment at the hospital and home infection risk was 19 and 11%, respectively.

### Mental health of our participants

The mean anxiety score was 5.36 (*SD* = 3.22), and the mean depression score was 8.35 (*SD* = 3.33). In our participants, 25% of them can be considered cases of anxiety (with HAD-A score at 8 or more) and 56% as for cases of depression (with HAD-D score at 8 or more). The percentage of cases having moderate to severe anxiety and depression symptoms were 6 and 26%, respectively, among all participants.

There’s a moderate to strong correlation between anxiety and depression (*r* = .69, *p* < .001). The participants with anxiety symptoms are also likely to have depressive symptoms.

### Comparing working and non-working family carers

Working family carers were significantly more depressed compared to non-working family carers (*M*_*working*_ = 8.71, *SD*_*working*_ = 3.36; *M*_*non-working*_ = 7.81, *SD*_*non-working*_ = 3.21; *t* (234) = 2.04, *p* = .04). However, no difference can be observed between working and non-working family carers in anxiety, *t* (234) = .62, *p* = .54. (See Table 2 for details).

**Table 2:** Descriptive Statistics and Comparisons between Case and Non-case on Anxiety and Depression (*N* = 236)

Subsample size	Anxiety			Depression		
	Case	Non-case	Test statistics	Case	Non-case	Test statistics
	58 (25%)	178 (75%)		133 (56%)	103 (44%)	
Demographic factor						
Age	58.50	64.04	*t*(234) = 2.48, *p* = .01	60.71	65.22	*t*(234) = 2.32, *p* = .02
Women	0.83	0.71	*χ*^2^(1) = 2.97, *p* = .09	0.75	0.73	*χ*^2^(1) = 0.17, *p* = .68
Year in caregiving	6.63	7.71	*t*(234) = 0.95, *p* = .34	7.38	7.53	*t*(234) = 0.15, *p* = .88
Psychosocial factor						
Perceived risk	3.10	2.54	*t*(234) = −4.43, *p* < .001	2.78	2.54	*t*(234) = − 2.19, *p* = .03
Perceived severity	3.54	3.06	*t*(234) = −3.46, *p* < .001	3.29	3.04	*t*(234) = − 2.04, *p* =. 04
Employment difficulties	0.36	0.35	*χ*^2^(1) = 0.01, *p* = .91	0.33	0.39	*χ*^2^(1) = 0.84, *p* = .36
Financial difficulties	0.34	0.29	*χ*^2^(1) = 0.71, *p* = .40	0.29	0.32	*χ*^2^(1) = 0.33, *p* = .57
External factor						
Protective supplies	3.55	3.83	*t*(234) = 2.72, *p* = .007	3.70	3.85	*t*(234) = 1.61, *p* = .11
Home infection risk	0.17	0.10	*χ*^2^(1) = 2.55, *p* = .11	0.13	0.10	*χ*^2^(1) = 0.54, *p* = .46
Center services suspended	0.40	0.21	*χ*^2^(1) = 8.21, *p* = .004	0.31	0.18	*χ*^2^(1) = 4.69, *p* = .03
Getting drugs from the hospital	0.45	0.22	*χ*^2^(1) = 11.51, *p* = .001	0.34	0.19	*χ*^2^(1) = 6.05, *p* = .01
Delayed treatment at the hospital	0.19	0.20	*χ*^2^(1) = 0.01, *p* = .91	0.23	0.16	*χ*^2^(1) = 1.82, *p* = .18
Protective measure						
Wearing mask	4.79	4.88	*t*(234) = 1.42, *p* = .15	4.85	4.87	*t*(234) = 0.44, *p* = .66
Hand hygiene	4.16	3.98	*t*(234) = −1.62, *p* = .11	4.11	3.92	*t*(234) = −1.91, *p* = .06
Social distancing	3.84	3.57	*t*(234) = −2.07, *p* = .04	3.62	3.66	*t*(234) = 0.40, *p* = .69
Healthy lifestyle	3.22	3.49	*t*(234) = 2.55, *p* = .01	3.27	3.62	*t*(234) = 3.92, *p* < .001
Mental health outcome						
Anxiety	9.55	4.00	*t*(234) = −17.07, *p* < .001	6.80	3.50	*t*(234) = −9.07, *p* < .001
Depression	11.74	7.25	*t*(234) = − 10.97, *p* < .001	10.70	5.32	*t*(234) = − 20.64, *p* < .001

Case and non-case were compared on anxiety and depression

### Correlation among predictors of mental health

Correlation coefficients among studied variables are shown in Table 3. Age was negatively correlated with perceived risk (*r* = −.23, *p* < .001), perceived severity (*r* = −.14, *p* < .05), hand hygiene (*r* = −.21, *p* < .01), anxiety (*r* = −.14, *p* < .05) and depression (*r* = −.23, *p* < .001); while it was positively correlated with healthy lifestyle (*r* = .19, *p* < .01). Being a woman was positively correlated with the practice of social distancing (*r* = .14, *p* < .05) and anxiety (*r* = .15, *p* < .05). The number of years in caregiving was negatively associated with their perceived sufficiency of protective supplies (*r* = −.17, *p* < .01).

**Table 3:** Correlation coefficients among studied variables

		1	2	3	4	5	6	7	8	9	10	11	12	13	14	15	16	17
1	Age																	
2	Women	−.07																
3	Year in caregiving	.11	−.03															
4	Perceived risk	−.23^***^	.06	−.04														
5	Perceived severity	−.14^*^	.05	−.02	.56^***^													
6	Employment difficulties	−.04	.08	−.09	.14^*^	.18												
7	Financial difficulties	−.02	−.01	−.01	.18^**^	.21^**^	.40^***^											
8	Protective supplies	−.09	−.06	−.17^**^	−.08	−.07	−.09	−.25^***^										
9	Home infection risk	−.02	−.06	.04	−.09	−.03	−.02	.03	.03									
10	Center services suspended	−.16^*^	.03	.00	.13^*^	.11	−.09	.04	−.09	−.09								
11	Getting drugs from the hospital	−.06	.08	−.02	.15^*^	.19^**^	.02	.17^**^	−.18^**^	.14	.14^*^							
12	Delayed treatment at the hospital	−.10	−.03	−.11	.06	.09	−.05	.03	.05	.06	.08	.15^*^						
13	Wearing mask	.01	.06	−.04	.00	.09	.10	.02	.18^**^	.03	−.01	−.04	.06					
14	Hand hygiene	−.21^**^	.04	.10	.15^*^	.11	.07	.01	−.08	.10	.01	.12	.06	.28^***^				
15	Social distancing	.07	.14^*^	.10	.08	.22^***^	.03	.04	−.05	.09	.07	.20^**^	.02	.17^**^	.25^***^			
16	Healthy lifestyle	.19^**^	−.05	.12	−.13^*^	−.19^**^	.05	−.05	−.04	.02	−.19^**^	−.04	−.05	.06	.28^***^	.09		
17	Anxiety	−.14^*^	.16^*^	−.11	.37^***^	.36^***^	.02	.16^*^	−.17^**^	.10	.19^**^	.33^***^	.04	−.04	.18^**^	.14^*^	−.27^***^	
18	Depression	−.23^***^	.10	−.10	.27^***^	.24^***^	−.03	.01	−.10	.01	.21^**^	.25^***^	.05	−.01	.14^*^	.06	−.30^***^	.69^***^

^*^ *p* < .05, ^**^ *p* < .01, ^***^ *p* < .001

Perceived risk and severity have positive associations with anxiety (for risk, *r* = .37, *p* < .001; for severity, *r* = .27, *p* < .001) and depression (for risk, *r* = .36, *p* < .001; for severity, *r* = .24, *p* < .001). Employment and financial difficulties were not correlated with anxiety and depression; suspension in getting centre services (with anxiety, *r* = .19, *p* < .01; with depression, *r* = .21, *p* < .01) and getting drugs from the hospital (with anxiety, *r* = .33, *p* < .001; with depression, *r* = .25, *p* < .001) did.

### Regression models predicting anxiety and depression

In the hierarchical regression models predicting anxiety (in Table 4), we have four blocks of variables, including demographic factors (age, as women, having full-time or part-time job, and year in caregiving), psychosocial factors (perceived risk and severity of COVID-19, employment and financial difficulties), external factors (sufficiency of protective supplies, home infection risk, suspension of center services, getting drugs from the hospital, and delayed treatment at the hospital), and protective measures (wearing a mask, hand hygiene, social distancing, and health lifestyle), and they were added to a previous model at each step. In Model 4, as women (ß = .12, *p* < .05), perceived risk (ß = .21, *p* < .01), perceived severity (ß = .19, *p* < .01), home infection risk (ß = .13, *p* < .05), and getting drugs from the hospital (ß = .19, *p* < .01) were positive predictors of anxiety, while healthy lifestyle negatively predicted anxiety (ß = −.25, *p* < .001). The model explained 36% of the total variance in anxiety.

**Table 4:** Standardized regression coefficients of Hierarchical Regression Models Predicting Anxiety and Depression

	Anxiety								Depression							
	M1		M2		M3		M4		M1		M2		M3		M4	
Demographic factor																
Age	−.12		−.04		−.03		.05		−.21	**	−.16	*	−.15	*	−.08	
Women	.15	*	.14	*	.12	*	.12	*	.08		.07		.05		.04	
Year in caregiving	−.09		−.10		−.12	*	−.12	*	−.08		−.08		−.10		−.08	
Psychosocial factor																
Perceived risk			.22	**	.21	**	.19	**			.16	*	.15		.13	
Perceived severity			.22	**	.19	**	.15	*			.14		.11		.07	
Employment difficulties			−.12		−.09		−.07				−.09		−.06		−.04	
Financial difficulties			.13		.06		.06				−.02		−.08		−.09	
External factor																
Protective supplies					−.11		−.08						−.09		−.08	
Home infection risk					.13	*	.11	*					.03		.01	
Center services suspended					.10		.08						.12		.09	
Getting drugs from the hospital					.19	**	.17	**					.18	**	.17	**
Delayed treatment at the hospital					−.05		−.05						−.03		−.03	
Protective measure																
Wearing mask							−.08								−.01	
Hand hygiene							.21	**							.16	*
Social distancing							.02								−.01	
Healthy lifestyle							−.25	***							−.27	***
*R*^2^	.05	**	.22	***	.30	***	.36	***	.06	**	.13	***	.19	***	.25	***
Δ *R*^2^			.17	***	.08	***	.06	***			.07	**	.06	**	.06	**

^*^ *p* < .05, ^**^ *p* < .01, ^***^ *p* < .001

*M1* Model 1, *M2* Model 2, *M3* Model 3, *M4* Model 4

Similar to the analysis on anxiety, the same blocks of variables were put in predicting depression. In Model 4, both getting drugs from the hospital (ß = .17, *p* < .01) and hand hygiene (ß = .16, *p* < .05) positively predicted depression, while healthy lifestyle (ß = −.27, *p* < .001) negatively predicted depression. The model explained 25% of the total variance in depression.

Comparing the models of anxiety and depression, psychosocial factors (including perceived risk and severity) accounted for the largest *R*^*2*^ change (17%) in explaining the variance of anxiety (see Model 2), while they only accounted for 7% of the variance of depression.

### Difference in features between the case and non-case of anxiety

We further compared the demographic differences between cases and non-cases of anxiety to check if demographic variables may have caused the noise. Cases were significantly younger than non-cases of anxiety, *t* (234) = 2.48, *p* = .01; *M*_*case*_ = 58.50, *M*_*noncase*_ = 64.04. There were no differences between cases and non-cases regarding their biological sex and year in caregiving.

In psychosocial factors related to the pandemic, cases reported higher perceived risk, *t* (234) = − 4.43, *p* < .001; *M*_*case*_ = 3.10, *M*_*noncase*_ = 2.54, and perceived severity, *t* (234) = − 3.46, *p* < .001; *M*_*case*_ = 3.54, *M*_*noncase*_ = 3.06. No difference was observed in terms of reported employment and financial difficulties between cases and non-cases.

Considering the external factors regarding the pandemic, cases reported having fewer protective supplies, *t* (234) = 2.72, *p* = .007; *M*_*case*_ = 3.55, *M*_*noncase*_ = 3.83. Cases were more likely to raise concerns about the suspension of centre services, *χ*^*2*^ (1) = 8.21, *p* = .004, and getting drugs from the hospital, *χ*^*2*^ (1) = 11.51, *p* = .001.

In terms of protective measures, cases practised social distancing more than non-cases, *t* (234) = − 2.07, *p* = .04; *M*_*case*_ = 3.84, *M*_*noncase*_ = 3.57, but they lived less healthy lifestyle than non-cases, *t* (234) = 2.55, *p* = .01; *M*_*case*_ = 3.22, *M*_*noncase*_ = 3.49. No difference can be observed in wearing masks and hand hygiene between the groups.

### Difference in features between case and non-case of depression

A similar pattern can be observed comparing case and non-case of depression as in anxiety. Considering demographics, cases were younger than non-cases, *t* (234) = 2.32, *p* = .02; *M*_*case*_ = 60.71, *M*_*noncase*_ = 65.22. No difference can be observed between cases and non-cases in terms of sex and year in caregiving.

Cases reported higher perceived risk, *t* (234) = − 2.19, *p* = .03; *M*_*case*_ = 2.78, *M*_*noncase*_ = 2.54, and severity, *t* (234) = − 2.04, *p* = .04; *M*_*case*_ = 3.29, *M*_*noncase*_ = 3.04, than non-cases, but were indifferent in terms of reported employment and financial difficulties.

In terms of external factors, cases reported more concern about center services suspension, *χ*^*2*^ (1) = 4.69, *p* = .03, and getting drugs from the hospital, *χ*^*2*^ (1) = 6.05, *p* = .01. There was no difference in the perceived sufficiency of protective supplies and the concern about home infection risk and delayed treatment at the hospital.

Considering protective measures, cases lived less healthy of lifestyles than non-cases, *t* (234) = − 3.92, *p* = .04; *M*_*case*_ = 3.27, *M*_*noncase*_ = 3.62. No difference can be observed in other protective measures.

## Discussion

This was the first study to our knowledge examining the effects of psychosocial factors, external factors, and preventive behaviours related to COVID-19 on the mental health of family carers of older adults in Hong Kong. Regarding our RQ1, we examined the prevalence of anxiety and depression symptoms among family carers between April and May 2020, which was 3 months after the first confirmed case of COVID-19 in Hong Kong. The figure was alarming and revealed the heavier burden and life demands in family carers of older adults than the general population. Comparing to the prevalence of a recent population study in Hong Kong [46], that used Patient Health Questionnaire-9 (PHQ-9) (19%) and the Generalized Anxiety Disorder-7 (GAD-7) (14%), we found a much higher prevalence of depression (56%) and anxiety (25%). The prevalence of depressive symptoms was, in fact, comparable to health care workers (about 56%) in Hong Kong in the pandemic period [47]. In some sense, family carers are health care workers who work at “home”, but would have been easily overlooked when the focus is often on the care recipient. Care for the carers is something to be reminded if the care is to be sustainable.

The predictors we included in the regression models were mostly pandemic-related. The model explained 36% of the variance of anxiety and 25% of the variance of depression, indicating the pandemic had a considerable impact on the mental wellbeing of family carers. As shown by the significant *R*^*2*^ change, psychosocial factors, external factors, and preventive measures were all predictive of the mental health outcomes.

Psychological variables, including perceived risk and severity, as in many health behaviour models (for example, the Health Belief Model and the Protection Motivation Theory) [48], were predictive of anxiety. Anxiety is one of the primary responses to the pandemic as the virus created much uncertainty in terms of risk of infection and the severity of symptoms if one contracted COVID-19 [49]. Therefore, the psychosocial factors were more predictive of anxiety comparing the *R*^*2*^ change contributed by psychosocial factors in explaining the variance of anxiety and depression (17% versus 7%, see Model 2). The care recipient is not only an object of the care; it is someone the family carer loves and has concerns for.

Collecting prescribed medications from the hospital increased anxiety and depression among family carers for the increased risk of exposure to the coronavirus. Yet, it is inevitable that family carers need to accompany the care recipient to out-patient department of hospitals for regular follow-up and medication. Added to the burden is the professional practice of prescriptions only for a patient who turned up for consultation. Family carers are fearful that one or one’s care recipient will contract the COVID-19 virus during their visit to this high-risk place in the pandemic [50], and contraction of the deadly virus (to the older adults), even in its slightest form, would mean isolation for the patient and the disruption of care to the older adults, and in a more serious case, mean an imminent painful death and a denial of a proper funeral. It is not surprising that visiting the hospital can be anxiety- and stress-provoking.

Although the government has imposed visiting restrictions in order to minimize nosocomial infection, it may not be easy to change the impression of the hospital being a place with a higher infection risk. Harnessing the power of telemedicine technology could be a way to mitigate the anxiety accompanying the hospital visits and minimize unnecessary visits. The Internet Hospital plus Drug Delivery (IHDD) platform [51], for example, is a novel service found to be efficient in minimizing the influx of patients with mild conditions in its pilot trial in Shenzhen, China. Prescriptions can be done online, while the patients can choose to get the drugs by self-pickup or drug delivery service. Online pharmaceutical care services can be an alternative or supplementary practice to resolve the concern in drug use while avoiding unnecessary hospital visits [52]. Adoption of these kinds of platforms can be helpful to family carers in the long run to alleviate their burden of transportation and worry of exposure to pathogens.

Anxiety could be functional from the health communication point of view. A healthy lifestyle was a strong protective factor of anxiety and depression in family carers. Even though our data could not tell whether the healthy lifestyle changed due to the pandemic, the relationship could be a reciprocal one. It is possible that anxiety and depression might increase the family carers’ hand hygiene behaviours, resulting in a more health-consciousness and healthy lifestyle. Our data show that more hand hygiene behaviours (maybe obsessive) are a common indicator for concerns among family carers in this pandemic. Studies have shown that the fear and anxiety provoked by the pandemic can be a functional one in increasing public health compliance [53, 54]. The take-home message for the public health policymakers would be to maintain the anxiety in public at an appropriate/optimal level through health communication. The public health measures may be better implemented if anxiety is maintained at a certain level by communicating the risk of the infection. However, the paradox is that when it is too much, it is detrimental to health and coping. As personal protective measures are predictive of one’s anxiety and depression [54–56], a behavioural checklist of these protective measures may be a reliable proxy for frontline practitioners to probe into family carers who are not expressive or educated enough to read into the inventory texts.

Regarding our RQ2, as expected, we observed a negative association between age and neurotic symptoms. In our sample, more than half of our participating family carers (59.3%) were older adults aged 60 or above. In line with the socioemotional selectivity theory [34], older people are better in regulating their emotions through selecting positive and emotionally meaningful information, which subsequently contribute to their mental wellbeing. For many carers, caregiving duties are the core of their daily living, it is reasonable to expect that they may try to derive meanings from these responsibilities [57]. It may become a strategy for them to cope with the burden and demands of the caregiving tasks [58]. Our results reveal the need to provide mental health care support to younger family carers, those with less caregiving experiences, and those working family carers who are challenged on both ends (work and family caregiving) during the pandemic time.

The current study has a number of limitations. Firstly, the non-probability sampling in our study might potentially result in biased estimates. Secondly, no causal relationship could be inferred from the cross-sectional design. Thirdly, we did not probe or record the actual frequency/intensity of the protective measures like mask-wearing and hand-washing, and therefore provided only rough rather than a fine estimate of the protective measures. Fourthly, caregiving for older adults in residential care would represent different challenges that this study did not capture. For example, no family visitors were allowed during certain periods, and this made older people in the under-staffed private nursing home in Hong Kong more susceptible to bed-ridden diseases. Fifthly, the caseness of depression or anxiety of the caregiver was determined by the inventories rather than the more valid form of clinical assessment.

## Conclusions

In conclusion, the study revealed a high prevalence of anxiety and depression symptoms among family carers of the elderly as compared to the general population in Hong Kong. Family carers have both psychosocial and pragmatic concerns in the COVID-19 pandemic. Telemedicine practices, communal dispensary, and pharmacy on delivery could be helpful in alleviating their anxiety and depression by reducing the risk of exposure to infection. Further studies are needed to examine the difference in their needs between older and younger family carers and between working and non-working family carers.

## Data Availability

The data that support the findings of this study are available on request from the corresponding author, MC. The data are not publicly available due to their containing information that could compromise the privacy of research participants.

## Abbreviations

HADS: Hospital Anxiety and Depression Scales
HAD-A: Hospital Anxiety and Depression Scales – Anxiety subscale
HAD-D: Hospital Anxiety and Depression Scales – Depression subscale
IHDD: Internet Hospital plus Drug Delivery
M: Mean
NGO: Non-government organization
SARS: Severe Acute Respiratory Syndrome
SD: Standard deviation
